# Editorial: Transforming academia for equity

**DOI:** 10.3389/fpubh.2026.1893043

**Published:** 2026-06-18

**Authors:** Linda A. Alexander, Jordan A. Fox, Tawara D. Goode, Sheldon Watts

**Affiliations:** 1 Association of Schools and Programs of Public Health, Washington, DC, United States; 2 Georgetown University National Center for Cultural Competence, Washington, DC, United States; 3 Robert Wood Johnson Foundation, Princeton, NJ, United States

**Keywords:** academic public health, antiracism, heath equity, institutional transformation in higher education, public health education

*Transformation can be defined as a metamorphosis—a radical change in a person, team, department, or organization with a goal of meaningful and lasting improvement*.

“*The very idea of transformation is appealing to many, as it carries with it a promise of real change that contributes to greater equity, justice, and sustainability, both locally and globally*” (1).

## Introduction

The Association of Schools and Programs of Public Health (ASPPH) remains committed to dismantling systemic racism and ending the cultural and structural conditions that enable injustice, hate, colonialism, and disparate morbidity and mortality. We see academic public health institutions as having a primary role in facilitating this change. As the “voice” of academic public health we currently serve 155 schools and programs of public health and are uniquely positioned to enhance the social justice mandates of public health through our direct impact and involvement in teaching, research, and service. ASPPH's long-standing efforts in this arena are reflected in the 2030 Strategic Plan for the association and in a published report entitled “Dismantling Racism and Structural Racism in Academic Public Health: A Framework” (2). The Framework recommendations are intended to serve as a blueprint for academic institutions to achieve inclusive excellence in the domains of teaching, research, and service. These domains were recently operationalized via a unique strategic partnership with the Robert Wood Johnson Foundation (RWJF), who funded ASPPH, seven academic institutions, and an organizational change partner to imagine, design, and implement transformative approaches to institutional change. These adaptive change strategies provide critical insights into the complexities, nuances, and opportunities involved in *Transforming academia for equity* and preparing a competent 21st-century public health workforce.

To maximize impact and reach the broad dissemination strategy for the transformative actions needed to achieve equity in the academy, this effort culminated the current Frontiers in Public Health Special Supplement: *Transforming academia for equity*.

## Transforming academia for equity

Transforming academia to advance equity in public health research will require radical change. Change is needed in the cultures of institutions of higher education (IHEs) including values, principles, policies, structures, practices, and resource allocation of their research portfolios and enterprises. Change is also needed in the cultural expectations of research faculty and teams including their mindsets, knowledge, skills, behaviors, attitudes, and capacity for adaptation. Transformation requires that IHEs reexamine, reimagine, redesign, and restructure all facets of research to align with what constitutes equity—not only conceptually—but in the sociocultural contexts of populations and communities that should benefit from public health research. Much is written in the literature that transformative organizational change is culture change. Culture influences people, disciplines, teams, departments—the overall environment in which research is conducted within schools and programs of public health. According to the Royal Society of London, “research culture encompasses all behaviors, values, expectations, attitudes and norms of research communities” (3). Culture and language are inextricably linked. Embracing cultural humility and linguistic competence as evidence-based practices is an essential component in the process of transformative change for inclusive excellence in the conduct of equitable public health research.

Academic public health is at a critical juncture. Persistent inequities in health outcomes, education, workforce representation, and institutional culture have underscored the limitations of traditional academic models that often fail to address the structural and historical forces that influence health and opportunity. At the same time, the current socio-political environment has intensified these challenges, with growing political attacks on public health institutions, restrictions on diversity, equity, and inclusion efforts, erosion of trust in science, and policy actions that threaten evidence-based practice and population health protections (4). Increasingly, there is recognition that advancing equity requires more than incremental reform or the addition of isolated diversity initiatives. Rather, it demands a fundamental reexamination of how public health knowledge is produced, taught, evaluated, and applied within academic institutions and community settings.

The articles included in this Research Topic, *Transforming academia for equity*, reflect this broader shift. Across diverse disciplinary, institutional, geographic, and cultural contexts, the authors contributing to this Research Topic examine how systems of power, historical inequities, and institutional practices continue to shape educational experiences, professional advancement, community engagement, institutional culture, and public health practice. At the same time, they offer innovative approaches to curricular transformation, mentorship, leadership, community partnership, institutional change, and equity-centered evaluation.

Trauma-informed approaches, while applied more broadly to health and human services, have more recently been embraced by institutions of higher learning (5). Several articles foreground the importance of historical and structural context in shaping educational experiences. Nhari et al. examine how intergenerational trauma influences ethics education and advocate for culturally grounded frameworks such as Ubuntu, while Chibambo et al. trace persistent inequities in Southern African higher education to colonial and apartheid legacies and call for greater integration of indigenous knowledge systems. Together, these contributions demonstrate that *Transforming academia for equity* requires deeper engagement with how knowledge is constructed, valued, and taught.

This argument is reinforced by Evidence for Action, a national program of the Robert Wood Johnson Foundation through its Ways of Knowing Initiative (6). This initiative aims to transform the health research system by promoting a broader understanding of how people interpret the world, advancing more inclusive and equitable approaches to generating and applying knowledge. This expanded view values lived experience, history, instinct, and other collective forms of wisdom alongside traditional forms of evidence generation (6). A traditional research paradigm deemphasizes the critical nature of a focus on anti-colonialism and anti-racism and ignores the role of systems and power in maintaining the status quo.

A similar attention to systems and power structures is evident in a mini review on global health initiatives by Stevanovic et al., where the authors argue that many programs continue to reinforce inequities due to high-income country dominance in academia and insufficient investment in local public health capacity. They advocate for models of global health collaboration that prioritize equitable partnerships and community leadership. A systems-oriented perspective is also reflected in Judson and Sharif's original research article, which applies Public Health Critical Race Praxis—a public health offshoot of Critical Race Theory—to police violence, reframing it as a public health issue that operates across structural, intergenerational, and community levels.

The “Dismantling Racism and Structural Racism in Academic Public Health” (2) framework provided important recommendations for curriculum changes to align with the spirit of transformation in the academy and underpinned much of the work of the RWJF-sponsored work by many institutions cited in this editorial. Several authors focus specifically on curricular transformation as a key mechanism for change.

In one article, Petersen describes a summer reading assignment used in a graduate public health course to teach students about structural racism and health equity. The structured exposure to these topics was found to support deeper learner understanding of social determinants of health and systemic racism, as well as greater engagement with complex public health issues. Similarly, Stief et al. describe the Penn, Social Systems, and the Community (PSSTC) program, a semester-long, asynchronous curriculum developed to prepare University of Pennsylvania Master of Public Health (MPH) students for community engagement and applied practice by examining how historical and systemic inequities shape public health. Evaluation findings indicated that students felt better prepared for applied practice experiences and public health work, while also reporting increased knowledge and practical strategies for engaging with diverse populations. Lassiter et al.'s original research article describes the integration of an antiracism curriculum across multiple core public health courses, revealing how a shared conceptual framework can be adapted across disciplines such as epidemiology, biostatistics, and policy. Rather than functioning as isolated curricular interventions, these approaches reinforce the idea that equity must be embedded across all aspects of training and education for public health.

Several articles emphasize that curricular innovation alone is insufficient, without overall institutional transformation. Whittaker and Montgomery's conceptual analysis distinguishes between willingness and readiness for change, arguing that meaningful transformation requires alignment across leadership, policies, and organizational culture. This distinction is critical, as it underscores the gap between intention and implementation that often characterizes equity efforts in academia. This theme is echoed in Joy's perspective article examining equity in academic professional promotion processes. Joy identifies systemic biases that disadvantage underrepresented groups, calls for structural reforms in evaluation and advancement criteria, and proposes strategies to reduce bias and improve equity in academic career advancement. Joshua et al.'s community case study on shared leadership models for diversity, equity, inclusion, and belonging demonstrates how collaborative governance structures can shift institutional culture by involving faculty, staff, and students in decision-making processes.

Extending this institutional perspective to the national level, Magaña et al.'s opinion article examines the Association of Schools and Programs of Public Health (ASPPH)'s role in advancing institutional excellence and equity across academic public health. The authors highlight initiatives such as Framing the Future 2030 and antiracism frameworks as mechanisms for promoting systems-level change in public health education, leadership, and workforce development. These contributions further emphasize that *Transforming academia for equity* requires changes in how institutions operate, not just in what they teach. Leadership, governance, and accountability structures are central to sustaining institutional transformation for equity.

Efforts to build institutional capacity are further illustrated by Cohen et al., who describe the Continuous Learning for Antiracist Culture Change (CLARCC) Fellowship, designed to strengthen antiracist pedagogy and leadership capacity among public health faculty and staff. Evaluation findings demonstrated increased participant capacity to apply antiracist frameworks while also highlighting challenges associated with sustaining long-term cultural change. At the organizational level, Levy et al.'s community case study describes the University of Memphis' data-driven approach to institutional transformation, using climate surveys, listening sessions, and focus groups to identify priorities and embed equity metrics into strategic planning, thereby supporting long-term sustainability. In both examples, it is clear that there remains a commitment at multiple levels, including those of leadership to transform a climate and culture to strengthen institutional capacity beyond that which is often derived from strategic planning exercises.

Mentorship and workforce development as key mechanisms for advancing equity in academia have not yielded an exhaustive list in academic public health. A conceptual analysis by Garcia et al. introduces a systemic mentorship model that reconceptualizes mentorship as a networked, relational process rather than a hierarchical, one-directional exchange, emphasizing shared responsibility and equity-centered relationships. The Mentoring of Students and Igniting Community (MOSAIC) program, described in complementary articles by Samari et al. and Grilo et al., demonstrates how structured mentorship can improve students' sense of belonging, academic confidence, and retention, while also highlighting strategies for sustaining such programs within institutional structures. In an Original research article, Wipfli et al. evaluate a community-based youth public health ambassador program, showing how engaging young people as leaders can strengthen both community engagement and the future public health workforce. Taken together, these studies suggest that *Transforming academia for equity* requires not only adapting curricula and policies, but also investing in people, relationships, and pathways. These findings align with broader scholarship emphasizing the importance of relational, equity-centered mentorship models and supportive training environments in strengthening workforce development and advancing institutional change (7, 8).

Building on the evolution of Community-Based Participatory Research (CBPR) over several decades since its initial framing, recent scholarship continues to emphasize the importance of experiential and community-engaged approaches to advancing equity-oriented public health education and practice (9). Three articles in this Research Topic reflect these themes in distinct yet complementary ways. Hasnain et al. describe the Interprofessional Approaches to Health Disparities (IAHD) course, in which students from multiple health disciplines collaborate with community partners using CBPR. The authors' findings demonstrate significant improvements in interprofessional collaboration and self-efficacy, underscoring the value of team-based, community-engaged education in addressing health inequities. Similarly, Raton-Hibanada et al.'s qualitative case study explores how gender equality, diversity, and inclusion (GEDI) principles are integrated into university community extension programs. Their findings highlight the opportunities and challenges of embedding equity principles within community-engaged initiatives, particularly in contexts shaped by cultural norms and resource constraints. Relatedly, Paulino et al.'s research on empathy in online counseling in Global South universities reveals the importance of culturally sensitive communication and demonstrates how digital environments can both hinder and reshape relational dynamics.

Several authors also explore pedagogical innovation and teaching practices. In a study examining award-winning public health educators, Weist et al. identify transformative, student-centered approaches as central to effective teaching, particularly when aligned with equity-focused frameworks. Separately, Bellamy and Sullivan's critique of biostatistics qualifying exams highlights how traditional assessment methods may reinforce inequities and fail to capture meaningful competencies, calling for more inclusive and reflective evaluation approaches. These articles underscore the need to rethink not only what is taught, but also the ways in which learning is assessed and valued.

Curricular reform into specific professional and disciplinary contexts beyond public health education are highlighted in three examples informed by both U.S. and international perspective. In their article, King et al. describe an inclusion health elective designed to prepare medical students to work with populations experiencing social exclusion and health inequities. The curriculum integrates service learning, problem-based learning, case-based learning, cultural humility, and immersive clinical experiences in low-resource settings to help medical students better understand the social determinants of health, structural inequities, and relational dimensions of care. MacCarthy et al.'s article examines efforts to strengthen LGBTQ+-affirming nursing education, identifying persistent gaps in existing curricula and proposing evidence-informed strategies for integrating LGBTQ+ health content, culturally responsive care practices, and inclusive instructional approaches into nursing training. In a systematic review, Libermann et al. analyze multicultural approaches in entrepreneurship education, pointing to the limited integration of intercultural and ethno-cultural perspectives into teacher training and program design. These three articles suggest that curricular transformation is most impactful when it is integrated, applied, and responsive to social, cultural, and structural context, rather than treated as an isolated or elective component of coursework.

The diversity of contexts represented in this Research Topic underscores that equity is not experienced uniformly, and that effective interventions must be culturally responsive. This includes recognizing whose knowledge is valued, how communication occurs, and how educational programs and institutional practices are designed to reflect the lived experiences of diverse populations.

A key cross-cutting theme within this Research Topic is the need to reframe academic evaluation and assessment through a racial and equity lens. Whether through the integration of equity metrics into strategic planning or critiques of traditional assessment systems, several contributions suggest that evaluation practices must align with equity goals and center the perspectives of communities and individuals most affected by structural inequities. This reflects a broader shift from viewing evaluation as a neutral process to understanding it as an integral component of equity work.

The valuable lessons contained within this Research Topic are noteworthy for their transferability both in domestic and global academic institutions. Moreover, the contemporary rhetoric that evokes the politicization of public health and is responsible for the erasure of structures that support equity and justice falls short of embracing the true mission of education. The transformative actions undertaken by these scholars acknowledge the procedures, policies, and systems required to prepare a competent 21st-century public health workforce capable of leading within increasingly complex public health environments that demand adaptive, interdisciplinary, and equity-centered solutions (10).

Together, the 25 articles included in this Research Topic demonstrate that transforming academic public health for equity requires sustained change across curriculum, mentorship, institutional culture, governance, and community engagement. As academic public health continues to evolve, the challenge will be to ensure that these efforts move beyond innovation to become embedded, scalable, and enduring practices. This Research Topic offers both a roadmap and a call to action, underscoring the importance of continued collaboration, reflection, and accountability in advancing equity across academic systems.
